# Effects of Exercise on Biomarkers in Health and Disease: Some New Insights with Special Focus on Extreme Exercise and Healthy Ageing

**DOI:** 10.3390/ijerph17061986

**Published:** 2020-03-18

**Authors:** Victor Machado Reis

**Affiliations:** Research Center in Sports Sciences, Health Sciences & Human Development, CIDESD, University of Trás-os-Montes and Alto Douro, 5000-801 Vila Real, Portugal; victormachadoreis@gmail.com

Health conditions associated with sedentary lifestyles continue to grow. Indeed, growing epidemics such as obesity and diabetes have been devastating for the human population. There have been improvements in medical care and new drugs, but neither seem to show signs that these tendencies can be stopped. More active lifestyles, namely including guided or non-guided physical activity, have been pointed out as strategies that are able to help in this battle. Past research has focused on the effects of exercise on health-related markers. Nevertheless, constant changes in demographics of major diseases, new treatment and therapeutics, and innovative proposals of exercise interventions all lead me to believe that the research on the overall contribution of exercise to public health has yet to peak. 

This Special Issue in the International Journal of Environmental Research and Public Health is devoted to recent findings on the “Effects of Exercise on Health-related Markers and Bioenergetics”. A number of valuable contributions are included in this Special Issue, which help to enlarge the body of knowledge that enables the design of effective practical interventions. As expected, the contributions cover a wide range of topics related to acute and chronic effects of exercise programs both in health and disease; highlighting the interplay between exercise, physical fitness, quality of life, and various health markers. The papers herein were produced worldwide, from the far East (Japan and Korea) to the West (USA), passing through Europe (Greece, Poland, Switzerland, Germany, Spain, Portugal) and South America (Brazil).

The scope of this Special Issue is broad enough to cover a variety of topics. I attempted to classify the papers into three groups which are summarized below. The first group includes seven papers which address the effects of extreme exercise on different biomarkers. By extreme exercise, I refer to both, long duration exercise (i.e., above 24 h runs) and high-intensity exercise. The second group includes seven papers which address exercise in obese or aged individuals. The remaining four papers vary in topics and are thus assigned to a non-specified group. The following sections present a sum of the main findings reported herein.

## 1. Health Biomarkers in Extreme Exercise

High-intensity exercise, namely some exercise programs typically labelled as extreme conditioning, have gain popularity in non-athletic populations, which has been the case of CrossFit^®^. Poderoso et al. [1] followed male and female crossfitters and found that six months of training increased basal levels of testosterone and lowered those of cortisol in men. They also described a positive adaptation of immune function biomarkers (CD8 T lymphocytes) in the fourth month of training. 

Extreme exercise is not limited to high-intensity short- to medium-duration exercise. Unusual long duration exercise also puts extreme stress on the human body. Acute kidney injury (AKI) is described as a relatively common complication of exercise, namely prolonged runs. Wolyniec et al. [2] found that typical urinary AKI markers were elevated after both 10 and 100 km runs, and concluded that there seems to be a physiological reaction rather than proof of kidney injury. However, their results support the use of neutrophil gelatinase-associated lipocalin and cystatin-C as the most valuable novel markers which can help in post-exercise AKI diagnosis.

Nikolaidis et al. [3] brought us a case-study of a 53-year-old experienced ultra-endurance athlete during his preparation for a 48 hour ultra-marathon race (3 month period). They report a pronounced reduction in fat-free mass change in body composition, especially during the first months of the training period, thereby highlighting the need for careful calorie intake calculation in these cases. The issue of nutritional intake and its effects on recovery post-high-intensity exercise was addressed by Araújo et al. [4] using an animal model. They supplemented rats with the inner bark of *Croton argyrophyllus* (Euphorbiaceae plant). This supplement is consumed in the northeast of Brazil and has antioxidant properties but was untested in the exercise setting. In this study, the supplementation showed the ability to partially reduce post-exercise biomarkers of oxidative stress at the plasma level, and in the muscle as well.

High-intensity exercise is often based on resistance exercise. Recently, the use of pre-exercise ischemia was found to change the adaptations of typical resistance training. Panza et al. [5] show that the pre-exercise ischemia enables a more pronounced hypotensive effect of resistance exercise in normotensive individuals.

Collectively, these five studies provide us with interesting insights on the effects of extreme exercise in health biomarkers. Engel et al. [6] also investigated the effects of eight weeks of functional “Tabata-type” high-intensity exercise on health-related markers, identifying small to moderate effect sizes for blood pressure and quality of life.

Another contribution on the high intensity exercise subject, this time focusing on the exercise program itself, namely on the combination of moderate vs. high intensity exercise, is also included in this Special Issue. Indeed, Byrd et al. [7] confirmed that typical moderate-intensity continuous exercise is not able to match the positive adaptations in physical fitness provided by its combination with high-intensity exercise. 

## 2. Exercise in Obesity and Ageing

Bariatric surgery has been vastly applied to obese people and its efficacy is usually dependent upon the adherence to healthy lifestyles post-surgery. Two interesting studies addressed the changes in cardiometabolic risk factors in this subject cohort for a 10-year post-surgery period. Pereira et al. [8] verified that the surgery induced more active lifestyles in the patients and improved both quality of life and cardiometabolic risk factors. On the other hand, the study by Almeida et al. [9] focused on the first-year post-surgery and followed patients from private vs. public health units. On admission, the patients from public units exhibited more elevated cardiometabolic risk factors compared with those from private units. Interestingly, both groups presented a similar risk one-year post-surgery. Exercise adherence in general seem to be largely dependent on motivation and this is also the case with obese individuals. Cabanillas-Cruz et al [10] conducted a study with obese individuals that underwent an exercise program in a hospital, describing that the main sources of motivation were weight loss and health improvement, while enjoyment during exercise was found to help patients’ engagement in the program.

With an increased worldwide life expectancy, special attention has been paid to exercise and ageing. Exercise programs often viewed as limited to young healthy individuals have been shown to improve health among aged populations, in health and in disease. The paper by Mendes et al. [11] compared the acute effects of high-intensity interval training vs. moderate-intensity continuous training on glycemic control in middle-aged and older patients with type two diabetes and under therapy with metformin and/or gliptins. The authors concluded that the higher intensity during treadmill exercise was safe and more effective in short-term glycemic control when compared with the lower intensity exercise. 

In aged individuals, overall body strength and mobility is extremely important in daily activities and help to foster an independent life. The original contribution from Bucht and Donath [12] investigated whether sauna yoga at a moderate temperature (50 °C) improved the flexibility, strength, balance, and quality of life of elders aged close to 70 years old throughout an eight-week period. They found that sauna yoga significantly improved flexibility as given by the chair sit-and-reach test.

Besides yoga, other Asian culture exercises have been proposed as appropriate for the aged. Im et al. [13] combined yoga and Korean dance to build an exercise program and tested its effects post-12 weeks in aged women. They reported improvements in balance, flexibility, muscle strength, serum growth hormone, dehydroepiandrosterone-sulfate, and estrogen in the experimental group.

Although evidence on the effects of exercise on the elderly are abundant, fewer studies have addressed the effects of detraining. Leitão et al. [14] analyzed the effects of a three-month detraining period following a nine-month physical training program in aged women. They found that the positive adaptations after the nine-month training period were partially impaired after the detraining. Some of those adaptations were still present, compared to baseline values of health biomarkers, such as cholesterol, resting heart rate, systolic blood pressure, and the six-minute walk test. Others biomarkers returned to baseline levels after the detraining period, which highlights the importance of exercise compliance in the elderly.

## 3. Other Original Contributions

Eating disorders tend to occur during adolescence, a period where individuals are prone to be highly influenced by mass media beauty patterns. Uchôa et al. [15] brought forward a study on this issue. They observed that mass media influenced the appearance of body dissatisfaction, and that this is a predictor of the risk to engage in eating disorders in both boys and girls. Obesity epidemics have led to the need to quantify the energy cost of daily activities. Caballero et al. [16] analyzed several of these activities and assessed predictors of its energy cost, concluding that relative heart rate reserve was the strongest predictor, with less than 10% estimation error when ascending or descending stairs.

The assessment of the energy cost of physical activity is reliable when expired gases are analyzed, especially at low-intensity exercises. Resistance exercise typically involves high intensities and therefore presents less-reliable energy cost estimation. Indeed, the assessment of the energy cost of resistance exercise is still poorly described. Reis et al. [17] contributed herein to the body of knowledge on the oxygen uptake (VO_2_) on-kinetics during low-intensity resistance exercise. They concluded that the absence of a true slow component indicates that it is possible to calculate low-intensity resistance exercise energy costs based solely on VO_2_ measurements, both in leg and arm exercises.

The establishment of biochemical markers that are able to quantify muscle fatigue and enable the control of training adaptations in sportsmen was addressed by the study of Nowakowska et al. [18]. While testing a number of blood markers, they concluded that aspartate aminotransferase, creatine kinase, lactate dehydrogenase, and creatinine levels, when analyzed together, could constitute a useful set of markers for monitoring recovery periods in soccer players.

## 4. Conclusions and Research Needs

We highlight that exercise adaptations in disease, namely in obese and aged individuals, seems to depend upon participants´ motivation, and exercise adherence should be one of—if not, the—most important requirement, when establishing programs for these subject cohorts. Whether this requirement is attainable with community group-exercise or if it warrants a personal training approach remains unclear and must be studied.

Extreme exercise, when conducted within general guidelines and in healthy subjects, seems to be safe; though it requires constant control with recovery tools (i.e., nutritional intake). However, due to its novelty, the dose–response is still poorly known. Hence, future research must seek more detail when examining high-intensity exercise programs, addressing the isolation of different training volumes, varied weekly frequencies, and, especially, exercise-recovery ratios within the exercise session itself.
